# Beyond attendance: reconsidering evidence and context in post-intensive care unit follow-up clinic models

**DOI:** 10.1186/s13054-025-05775-z

**Published:** 2025-11-27

**Authors:** Tuhin Mistry, Abhijit Sukumaran Nair

**Affiliations:** 1 Department of Anaesthesiology and Perioperative Care, Ganga Medical Centre & Hospitals Pvt Ltd, Coimbatore, India; 2 Department of Anaesthesiology, Ibra Hospital, Ibra, Sultanate of Oman

We read with great interest the systematic review by Chatterjee et al. that explored models and barriers of post-intensive care unit (ICU) follow-up clinics [1]. The authors provided a valuable overview of different approaches—hospital-based, hybrid, and telehealth/home models—and highlighted the key structural features such as team composition, visit frequency, and screening tools. Their use of the Consolidated Framework for Implementation Research adds further depth. However, a few points deserve clarification to prevent over-interpretation, especially regarding attendance rates and the relevance of findings to low- and middle-income countries (LMICs).

## Attendance does not equal effectiveness

The review reports mean attendance rates of 51.9% (in-person), 59% (hybrid), and 88.7% (telehealth/home) [1]. These numbers are simple averages from studies that differed markedly in design and sample size, and only three studies contributed to the telehealth group. Without adjusting for factors such as patient population, referral pattern, socioeconomic background, or access to technology, these averages may give the misleading impression that telehealth is superior. A weighted analysis that considers the number of patients in each study, or a sensitivity analysis excluding incomplete datasets, would provide more reliable comparisons.

## Clinic form versus function

The authors noted that evidence on patient outcomes was mixed. For example, a French randomized trial found that a multidisciplinary follow-up led by intensivists worsened 12-month quality of life compared with usual care [2]. This finding reminds us that the benefit of a clinic depends more on what it delivers and when interventions are provided, rather than where the clinic is held. The focus should therefore shift toward understanding the essential elements that aid recovery—such as physical rehabilitation, medication review, mental-health screening, and family support—and evaluating which combinations work best.

## Relevance to resource-limited settings

Although the review briefly mentions LMICs, most included studies were from high-income countries and published only in English [1]. This may have excluded evidence from regional journals or locally adapted, low-cost models [3]. Therefore, recommending hybrid clinics for LMICs may be premature. A more balanced statement could be: “Hybrid approaches appear promising, but evidence from LMICs regarding their feasibility and cost-effectiveness is still lacking. Local pilot projects tailored to available resources are needed.”

## Assessment of study quality

The review states that nearly all observational studies had a low risk of bias. This seems inconsistent with the typical limitations of service-evaluation studies, which often lack control groups or blinding. It would be helpful to clarify which domains of the Joanna Briggs Institute (JBI) checklist were considered “low risk” and whether important factors like confounding and selection bias were included [4]. To conclude, Chatterjee et al. provided a useful summary of how post-ICU follow-up clinics are structured. The next step is to determine which components truly aid recovery—who should deliver them, when they should occur after discharge, and how they influence patient-centred outcomes. Until stronger evidence is available, attendance rates or clinic formats should not be equated with effectiveness, particularly when applying findings to resource-limited settings.

## Data Availability

No datasets were generated or analysed during the current study.
